# Kir3 channel ontogeny – the role of Gβγ subunits in channel assembly and trafficking

**DOI:** 10.3389/fncel.2014.00108

**Published:** 2014-04-14

**Authors:** Peter Zylbergold, Rory Sleno, Shahriar M. Khan, Ashley M. Jacobi, Mark A. Belhke, Terence E. Hébert

**Affiliations:** ^1^Department of Pharmacology and Therapeutics, McGill UniversityMontréal, QC, Canada; ^2^Integrated DNA Technologies, Inc., CoralvilleIA, USA

**Keywords:** Kir3 channels, G proteins, G protein-coupled inwardly-rectifying potassium channels, Gβγ subunits, channel assembly

## Abstract

The role of Gβγ subunits in Kir3 channel gating is well characterized. Here, we have studied the role of Gβγ dimers during their initial contact with Kir3 channels, prior to their insertion into the plasma membrane. We show that distinct Gβγ subunits play an important role in orchestrating and fine-tuning parts of the Kir3 channel life cycle. Gβ_1_γ_2_, apart from its role in channel opening that it shares with other Gβγ subunit combinations, may play a unique role in protecting maturing channels from degradation as they transit to the cell surface. Taken together, our data suggest that Gβ_1_γ_2_ prolongs the lifetime of the Kir3.1/Kir3.2 heterotetramer, although further studies would be required to shed more light on these early Gβγ effects on Kir3 maturation and trafficking.

## INTRODUCTION

Kir3 channels were first discovered in the context of the K_ACh_channel (Kir3.1/Kir3.4), activated by the muscarinic acetylcholine receptor expressed in the heart. Logothetis et al. (1987) first noted that hyperpolarization of chick embryonic atrial cells via the iKACh depended on Gβγ but not Gα subunits. It was later proposed that Gβγ acted on Kir3 through direct interactions with the channel (Huang et al., 1995; Inanobe et al., 1995; Krapivinsky et al., 1995b) and that different interacting domains underlie basal and agonist-induced activation. Specifically, it was shown that Leu339 on Kir3.4 and its counterpart Leu333 on Kir3.1 were critical for receptor-stimulated currents and that mutation of these residues to Glu completely abrogated channel activation (He et al., 1999). Surprisingly, these mutations did nothing to basal current, suggesting that distinct regions on the channel allowed Gβγ to facilitate basal activity. It was shown that both the N- (aa 34–86) and C-termini of Kir3.1 could bind Gβγ and that there was in fact two distinct binding domains on the C-terminus (aa 318–374 and aa 390–462), conferring greater Gβγ binding when Kir3.1 was part of the tetrameric channel (Huang et al., 1997). They also showed that the N- and C-termini of Kir3 channels interacted with each other and that when the C-terminus of Kir3.1 interacted with either the N-terminus of Kir3.1 or Kir3.4, substantial increases in Gβγ binding were observed (Huang et al., 1997). It was later found that mutation of single residues His57 and Leu262 on Kir3.1 (His64 and Leu268 on Kir3.4, respectively) were sufficient to reduce Gβγ activation of the channel complex (He et al., 2002). Glutathione-S-transferase-pull down experiments and competition assays demonstrated critical Gβγ binding domains on the Kir3.2 subunit. Using Kir3.1 and Kir3.2 as templates, Ivanina et al. (2003) confirmed that similar regions of the N-terminus of the two subunits bound Gβγ, whereas significant differences were found at the level of the C-terminus. Consistent with Huang et al., (1997) Kir3.1 revealed two distinct Gβγ binding domains on its C-terminus, one proximal and one distal, while the Kir3.2 subunit possessed only one C-terminal binding site at its most distal end (Ivanina et al., 2003). Interestingly, mutation of these C-terminal sites on the Kir3.1 subunit, both proximal and distal, did little to the binding of Gβγ to the channel, but dramatically altered the current characteristics, suggesting these interacting domains were more involved in channel gating dynamics rather than Gβγ binding (Ivanina et al., 2003).

It was initially thought that Kir3 channels, like other effectors for G protein-coupled receptors (GPCRs), were trafficked independently of their cognate receptors, G proteins and auxiliary proteins to the plasma membrane. According to this model, functional interactions between signaling proteins would occur only upon receptor activation. However, this simple model does not account for the speed and specificity observed in signal transduction (Riven et al., 2006), and recent evidence suggests the existence of pre-assembled macromolecular signaling complexes containing Kir3 channels built before reaching the cell surface [(Nikolov and Ivanova-Nikolova, 2004; David et al., 2006; Rebois et al., 2006; Riven et al., 2006; Robitaille et al., 2009), reviewed in (Doupnik, 2008; Zylbergold et al., 2010)].

The notion of Kir3 signaling complexes arose initially from studies focusing on the interaction between D2- and D4-dopamine receptors, and Kir3 channels. We showed that the Kir3 channel could be co-immunoprecipitated in HEK 293 cells with either the D2- or D4- dopamine receptor, and that this interaction required the presence of Gβγ as shown by sequestration of Gβγ by the carboxy-terminal domain of GPCR kinase 2 (GRK2, βARKct; Lavine et al., 2002). We also showed that Kir3 channels interacted with their cognate G proteins well before reaching the cell surface (Rebois et al., 2006). These interactions could be observed with Kir3.1, in the absence of expressed partner subunits, implying that this interaction occured in the endoplasmic reticulum (ER; Robitaille et al., 2009). Using total internal reflection fluorescence (TIRF) microscopy combined with fluorescence resonance energy transfer (FRET), Riven et al. (2006) revealed that complexes of Kir3 and heterotrimeric G proteins exist at rest and that conformational changes in the channel facilitated opening of the channel gate. Furthermore, cell-surface interactions between Kir3, Gβγ, and the δ-opioid receptor (DOR), as detected by bioluminescence resonance energy transfer, have been described, supporting the notion that Kir3 complexes are stable in their journey from biosynthesis to functionality (Richard-Lalonde et al., 2013). Interactions between gamma-aminobutyric acid-B receptors and Kir3 channels have also been observed using an array of techniques (Fowler et al., 2007; Ciruela et al., 2010), with evidence suggesting that these interactions also occur soon after biosynthesis in the ER (David et al., 2006). Data suggesting that such complexes exist *in vivo* was first described by Nikolov and Ivanova-Nikolova (2004) whereby using rat atrial cardiomyocytes, they co-immunoprecipitated G proteins, GRKs, PKA, and protein phosphatases PP1/2, among others, with the KACh channel, indicating the presence of a highly coordinated complex for regulating cardiac excitability. Gβγ has been demonstrated to regulate many effectors [reviewed in (Khan et al., 2013)]. Early signaling complex formation has been observed for other effectors downstream of Gβγ signaling as well. Adenylyl cyclase has also been observed to stably associate with the β_2_AR (Lavine et al., 2002), with this interaction likely occurring concurrently with biosynthesis of the enzyme (Dupré et al., 2007). The existence of such complexes during receptor and effector biosynthesis along with the fact that Gβγ interacts with multiple signaling partners in the ER suggests that these are organizational events related to the specificity of cellular signaling, and places Gβγ subunits in a good position for acting as a central organizer of signalosome regulation and stability (Dupré et al., 2009; Zylbergold et al., 2010).

The role of Gβγ subunits in channel gating is well established. This article will explore the role of Gβγ dimers during their initial contact with Kir3 channels. We suggest that distinct Gβγ subunits play an important role in orchestrating and fine-tuning parts of the Kir3 channel life cycle.

## MATERIALS AND METHODS

### REAGENTS

Blasticidin, hygromycin, Dulbecco’s Modified Eagle’s Medium (DMEM), fetal bovine serum (FBS), penicillin/streptomycin, and tetracycline were from Wisent (St-Bruno, QC, Canada). Lipofectamine 2000 was obtained from Invitrogen (Burlington, ON, Canada). Anti-FLAG M2 affinity agarose beads were from Sigma–Aldrich (St. Louis, MO, USA). Bradford reagent, acrylamide and polyvinylidene fluoride (PVDF) membranes were obtained from Bio-Rad (Mississauga, ON, Canada). Enhanced chemiluminescence (ECL) Plus reagent was obtained from Perkin Elmer (Woodbridge, ON, Canada). The following primary antibodies were used: rabbit anti-Kir3.1 (Alomone Labs, Jerusalem, Israel), mouse anti-HA and mouse anti-myc (Covance, Princeton, NJ, USA), rabbit anti-FLAG (Sigma–Aldrich, St. Louis, MO, USA), mouse anti-glyceraldehyde 3-phosphate dehydrogenase (GAPDH) (Ambion, Burlington, ON, Canada), rabbit anti-Gβ_4_ (Santa Cruz, Dallas, TX, USA), mouse anti-Na^+^/K^+^-ATPase (Sigma–Aldrich, St. Louis, MO, USA), and mouse anti-β-tubulin (Invitrogen, Burlington, ON, Canada). The rabbit anti-Gβ_1_ antibody was a generous gift from Dr. Ron Taussig (UT Southwestern Medical Center). The following secondary antibodies were used: goat-anti-rabbit IgG (H+L) conjugated to Alexa488 (Invitrogen, Burlington, ON, Canada), goat-anti-mouse IgG (Fab specific) conjugated to peroxidase and goat-anti-rabbit IgG (whole molecule) conjugated to peroxidase (Sigma–Aldrich, St. Louis, MO, USA). PermaFluor aqueous mounting medium, bovine serum albumin and coverslips were obtained from ThermoScientific (Waltham, MA, USA). siRNAs targeted against Gβ_1_, Gβ_4_, Gγ_2_, Gγ_4_, Gγ_5_, and Gγ_7_ were purchased from Dharmacon (Ottawa, ON, Canada). Primetime qPCR 5′ nuclease assays were provided by Integrated DNA Technologies (Coralville, IA, USA). Unless otherwise stated, chemicals were of reagent grade and were purchased from Sigma–Aldrich (St. Louis, MO, USA).

### CLONING AND GENERATING THE INDUCIBLE CELL LINE

A previously developed inducible expression system was used to study pulses of Kir3.1 channels as they transit through biosynthetic pathways (Zylbergold et al., 2013). Briefly, extracellularly FLAG-tagged Kir3.1 was subcloned into the pcDNA5-FRT-TO inducible vector using BamHI and NotI restriction sites. The pcDNA5-FRT-TO-FLAG-Kir3.1 (2 μg) was co-transfected with the pOG44 recombinase gene (8 μg) into Flp-In^TM^ T-Rex^TM^ 293 parental cells in T75 flasks using Lipofectamine 2000 to generate inducible FLAG-Kir3.1 stables. 48 h post-transfection, cells were passaged and plated into new T75 flasks using fresh media (DMEM supplemented with 5% FBS and 1% penicillin/streptomycin – complete media) containing the selection antibiotics blasticidin (5 μg/mL) and hygromycin (200 μg/mL). Cells were grown to confluency and then used as required for subsequent experiments.

### INDUCTION AND TRANSFECTION PROTOCOL

Inducible FLAG-Kir3.1 stable cells were plated in T75 flasks, allowed to grow for 48–72 h and subsequently transfected with 2 μg of Kir3.2-MYC, FLAG-Gβ_1_, HA-Gγ_2_, or various siRNAs (depending on the experiment) using Lipofectamine 2000 (1 μg:2 μL ratio of DNA:Lipofectamine 2000; 6.25 nM:1 μL siRNA:Lipofectamine 2000, as per manufacturer’s protocol) in DMEM (0% FBS, 0% penicillin/streptomycin). Transfection media was then removed and replaced with complete media 5 h later. 24 h post-transfection, cells were induced with 1 μg/mL of tetracycline in DMEM for 30 min at 37°C, washed three times in DMEM and treated with 5 μg/mL of cycloheximide (CHX) in DMEM for 1 h at 37°C. Following CHX treatment, cells were washed twice in DMEM, and then incubated at 37°C in complete media supplemented with blasticidin for varying periods of time as needed for subsequent experiments.

### IMMUNOPRECIPITATION

Cells were plated in T75 flasks 48–72 h before transfection, co-transfected with 2 μg of Kir3.2-MYC or FLAG-Gβ_1_, HA-Gγ_2_, and then induced for Kir3.1-FLAG expression, 24–48 h post-transfection as described above. Transfected cells were washed twice with cold 1X phosphate buffered saline (PBS), harvested in 5 mL of cold 1X PBS, pelleted by centrifugation at 2000 rpm for 8 min at 4°C, and subsequently resuspended in a buffer containing 5 mM Tris pH 7.4, 2 mM EDTA, with protease inhibitor cocktail (10 μg/mL trypsin inhibitor, 5 μg/mL leupeptin, 50 μg/mL benzamidine). Samples were then subjected to two 10 s bursts with a polytron homogenizer on ice. Debris and unlysed cells were spun down for 5 min at 1000 rpm (4°C), and the supernatant was then fractionated by a 20 min centrifugation step at 16000 rpm (4°C) to pellet down the membranes. Pelleted membranes were resuspended in solubilization buffer (75 mM Tris pH 8, 2 mM EDTA, 5 mM MgCl2, 0.5% dodecylmaltoside, protease inhibitor cocktail) and incubated overnight with rotation at 4°C. The following day, membrane samples were spun for 5 min at 10,000 rpm (4°C) in order to remove unsolubilized membranes, resuspended in solubilization buffer and then quantified using the Bradford technique (Bio-Rad). In order to immunoprecipitate FLAG-tagged Kir3.1, 500 μg of sample was added to 25 μL of washed anti-FLAG M2 affinity agarose beads and incubated on a rotator overnight at 4°C. The following day, samples were washed three times [2 min at 1600 rpm (4°C)] in solubilization buffer supplemented with 300 mM potassium chloride. Bound proteins were eluted off of beads using 60 μL of Tris-buffered saline (TBS) solution (50 mM Tris pH 7.4, 150 mM sodium chloride) containing competitive FLAG peptide (3 μL/100 μL TBS) for 20 min on a 4°C rotator. Eluted proteins were spun down for 2 min at 1600 rpm (4°C) and then the supernatant was collected and mixed with 4X sample buffer (62.5 mM Tris, 16.3% glycerol, 2% SDS, 5% β-mercaptoethanol, 0.025% bromophenol blue) in a 1:4 ratio. Samples were then subsequently analyzed by western blot.

### WESTERN BLOTTING

For western blotting experiments, 60 μL of samples obtained following immunoprecipitation (IP) and 50 μg of samples from total lysate fractions were loaded on 8% polyacrylamide gels. Gels were electrophoresed for 90 min at 130 V and then transferred to PVDF membranes (Bio-Rad, Mississauga, ON, Canada) for 1 h at 100 V. Following transfer, PVDF membranes were blocked in 5% milk/TBS-containing 1% Tween (TBST) for 1 h at room temperature (RT). Membranes were probed with the primary antibodies as needed [anti-Kir3.1 (1/5000); anti-c-Myc (1/5000); anti-HA (1/5000); anti-Gβ_1_ (1/5000); anti-Gβ_4_ (1/400); anti-GAPDH (1/5000); anti-β-tubulin (1/5000); anti-Na^+^/K^+^-ATPase (1/5000)] in 5% milk/TBST overnight on a 4°C rotator. The following day, membranes were washed three times in TBST for 10 min, incubated in the appropriate secondary antibody [anti-rabbit or anti-mouse HRP (1/20000)] in 5% milk/TBST for 1 h at RT, and then subsequently washed again three times in TBST for 10 min. ECL Plus reagent was then added to the membranes and chemiluminescence was detected using a standard film developer.

### IMMUNOFLUORESCENCE

Cells plated on coverslips in six well plates 48–72 h before transfection were transfected with 300 ng of Kir3.2-MYC or empty vector (pcDNA3), and then induced for Kir3.1-FLAG expression 24–48 h later. At the time of harvest, cells were washed once in 1X PBS pH 7.4 and then fixed in 2% paraformaldehyde (PFA) for 10 min at RT. Cells were then washed three times in 1X PBS pH 7.4 to remove remaining PFA and then blocked in blocking buffer (1X PBS supplemented with 2% BSA) for 1 h at RT. Immediately following blocking, 200 μL blocking buffer with diluted rabbit anti-FLAG antibody (1/250) was applied to each coverslip and incubated overnight at 4°C. The following day, cells were washed three times in 1X PBS pH 7.4 and incubated with 200 μL blocking buffer supplemented with anti-rabbit Alexa488 (1/500) for 1 h at RT in the dark. Cells were then washed three times in 1X PBS pH 7.4 and coverslips were mounted on glass slides using PermaFluor Aqueous Mounting Medium and then imaged using a Zeiss LSM510 confocal microscope.

### REVERSE TRANSCRIPTION AND qPCR

For assessment of knockdown efficiency of Gγ subunits, total RNA was isolated from siRNA transfected inducible FLAG-Kir3.1 stable cells with TRI reagent using a modified RNA isolation protocol from Ambion. 2 μg of total RNA was reverse transcribed using a Moloney Murine Leukemia Virus Reverse Transcriptase (MMLV-RT, Promega) reaction assay as per the manufacturer’s protocol. Reverse transcribed cDNA was subject to qPCR using Custom Primetime qPCR 5′ Nuclease assays (IDT) as per the manufacturer’s protocol in a Corbett Rotorgene 6000 qPCR instrument. Percentage knockdown efficiencies were calculated using C_t_values obtained from the qPCR reaction that were subsequently analyzed using the 2^-△△Ct^ method, using the levels of hypoxanthine-guanine phosphoribosyltransferase (HPRT) as the housekeeping gene.

## RESULTS

**FIGURE 1:**
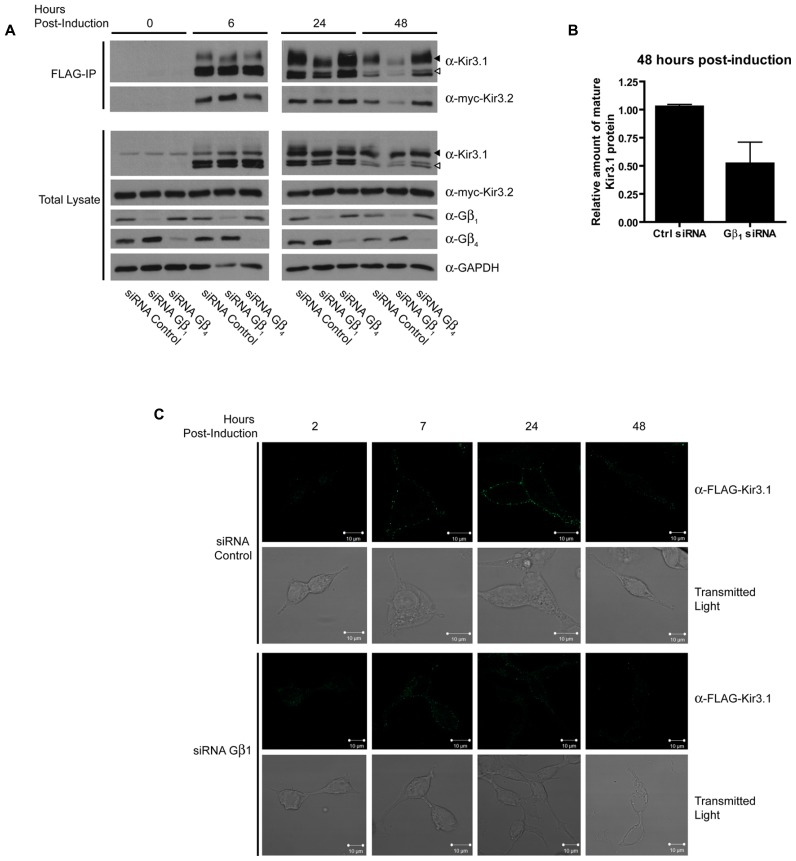
**Gβ_1_ but not Gβ_4_ knockdown reduces stability of Kir3.1. (A)** Flp-In-Trex-FLAG-Kir3.1 cells transfected with myc-Kir3.2 and Gβ_1_, Gβ_4_, or control siRNA were induced to express FLAG-Kir3.1 with 1 μg/mL of tetracycline for 30 min and then chased for 0, 6, 24, or 48 h. Upper panel: cells were lysed and FLAG-Kir3.1 was immunoprecipitated using an α-FLAG antibody. Samples were then analyzed by western blot to detect levels of FLAG-Kir3.1 and myc-Kir3.2. Lower panel: total cell lysates showing expression of Kir3.1, Kir3.2, Gβ_1_, Gβ_4_, and GAPDH (loading control). Results are representative of three independent experiments. White arrow indicates immature channel proteins while black arrow indicates correctly processed Kir3.1. **(B)** Densitometric analysis of data from experiments conducted above. The trend is clear and statistically significant at *p* < 0.1. **(C)** Flp-In Trex FLAG-Kir3.1 cells were plated on glass coverslips. The cells were transfected with siRNA targeted against Gβ_1_ and myc-Kir3.2. Cells were subsequently induced to express FLAG-Kir3.1 with 1 μg/mL of tetracycline for 30 min followed by chase times of 2, 7, 24, or 48 h. Cells were fixed with 2% PFA and then labeled (top panels) with rabbit α-FLAG followed by an α-rabbit secondary antibody conjugated to Alexa488 or shown using transmitted light (lower panels). Slides were imaged using a Zeiss LSM510 confocal microscope. Results are representative of a single experiment with several independent fields of cells.

**FIGURE 2:**
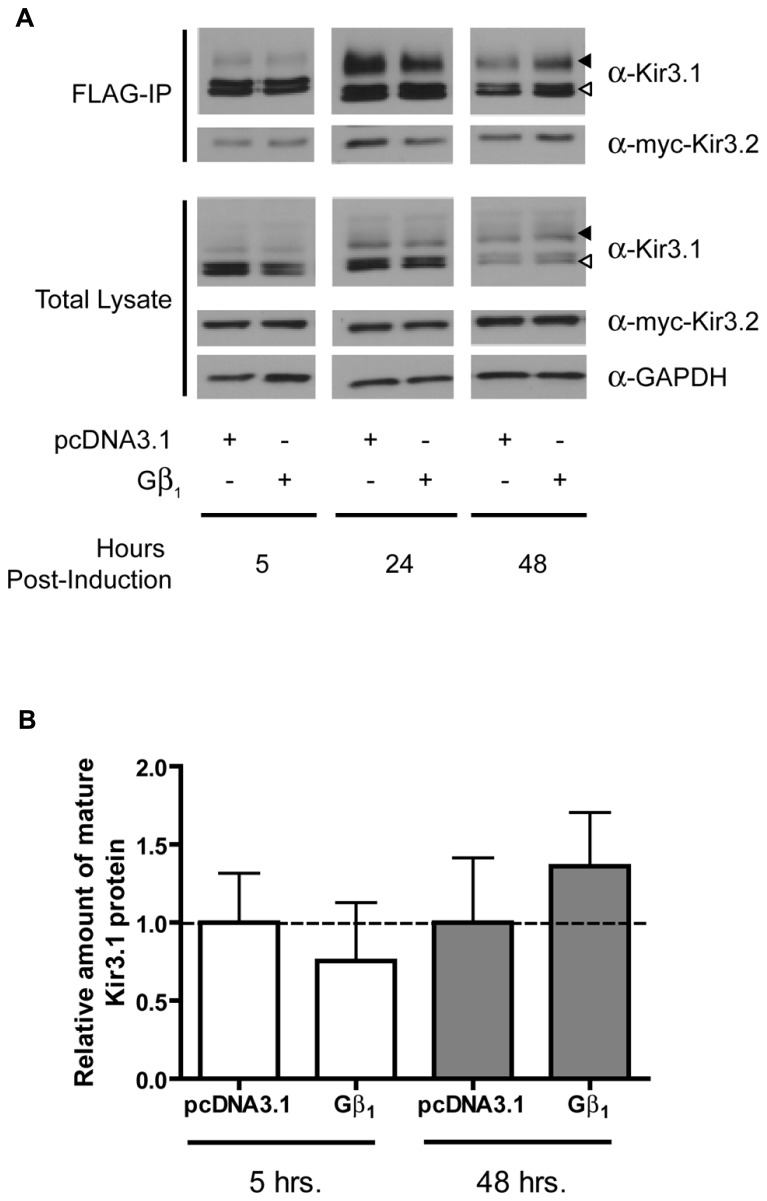
**Overexpression of Gβ_1_ stabilizes Kir3.1. (A)** Flp-In Trex FLAG-Kir3.1 cells co-expressing Gβ_1_ and myc-Kir3.2 were induced to express FLAG-Kir3.1 with 1 μg/mL of tetracycline for 30 min followed by chase times of 5, 24, or 48 h. Cells were lysed and FLAG-Kir3.1 was immunoprecipitated as described above. Upper panel: samples were analyzed by western blot to detect levels FLAG-Kir3.1 and myc-Kir3.2. Lower panel: total cell lysates showing expression of Kir3.1, Kir3.2, and GAPDH (loading control). Results are representative of two independent experiments. White arrow indicates immature channel proteins while black arrow indicates correctly processed Kir3.1. **(B)** Densitometric analysis of results obtained above. There is a trend in the effects of Gβ_1_ expression at 48 h, which is reflected in the western blots.

In order to assess the role of Gβγ subunits in the maturation and stability of Kir3.1 channels, we combined our Kir3.1 inducible expression system with RNA interference to knockdown different Gβ and Gγ subunits. Cells co-expressing Kir3.2 and control or different Gβ siRNAs were induced for 30 min with tetracycline and residual leak expression was silenced by a brief treatment with CHX. After washout, Kir3.1 maturation was followed at different time points. The advantage of our system is that it allows for a pulse of channel expression, at physiological protein levels, to mature and traffic to the plasma membrane without saturating the biosynthetic or quality control machinery (Zylbergold et al., 2013). After 6 h, it was observed that sufficient Kir3.1 is available for immunoprecipitation although the immature form is seen immediately after the 30 min pulse (**Figure 1A**; compare times 0 and 6 h in the FLAG IP and Total Lysate blots). The pulse of Kir3.1 expression also indicates that the channel reached maximum levels at 24 h post-induction and then levels subsequently begin to decline. Initial experiments demonstrated the selectivity of the siRNAs for Gβ_1_ and Gβ_4_ and confirmed efficient silencing of their respective endogenous transcripts in HEK 293 cells as assessed by the depletion of their gene products at the protein level (**Figure 1A**; Total Lysate). Although both Gβ_1_ and Gβ_4 _ are able to modulate channel function in response to GPCR stimulation (Lei et al., 2000), they are not identical with respect to their effects on channel maturation. In the presence of Kir3.2, Kir3.1 adopts a mature glycosylation phenotype and is trafficked to the cell surface (**Figures 1A–C**). When we knocked down Gβ_1_, there was little effect on the initial interactions between Kir3.1 and Kir3.2 and early maturational events still occurred. However, the amount of mature and immature channel both decline by 24 h in the absence of Gβ_1_, suggesting that the holo-channel is less stable and is likely targeted for early degradation. This observation is supported by the fact that much less channel appears on the cell surface when Gβ_1_ was knocked down, as detected using confocal microscopy (**Figure 1C**). The punctate staining of surface Kir3.1 is consistent with more physiological levels of the channel and again represents one of the advantages of our expression system. The loss of Gβ_1_ results in a more rapid life cycle for the channel. Knockdown of Gβ_1_ had no effect on levels of Kir3.2 (**Figure 1A**, Total Lysate), possibly representing either a selective effect on Kir3.1 or a reflection of the fact that bulk expression of Kir3.2 may escape quality control. Future experiments with inducible Kir3.2 constructs may help resolve this issue. Unlike Gβ_1_, knockdown of Gβ_4_ had no effect on the stability of the holo-channel, demonstrating a distinct role in channel function that may be restricted to activating the channel in response to receptor stimulation (**Figure 1A**). However, we did observe increased amounts of mature Kir3.1 when Gβ_4_ was silenced. This may suggest that remaining Gβ_1_ is now more able to interact with the channel in the absence of a competitor. Our data points to a clear demarcation between the functions of Gβ_1_ and Gβ_4_ with respect to Kir3.1 stability and trafficking. Consistent with our RNA interference data, we also noted that overexpression of Gβ1 resulted in holo-channels that were more stable at 48 h post-induction (**Figure 2**). Loss of the Gβ_4_ subunit also had no effect on levels of Kir3.2 in total cell lysates (**Figure 1A**, Total Lysate) suggesting that their effects are specific for Kir3.1 as well.

**FIGURE 3:**
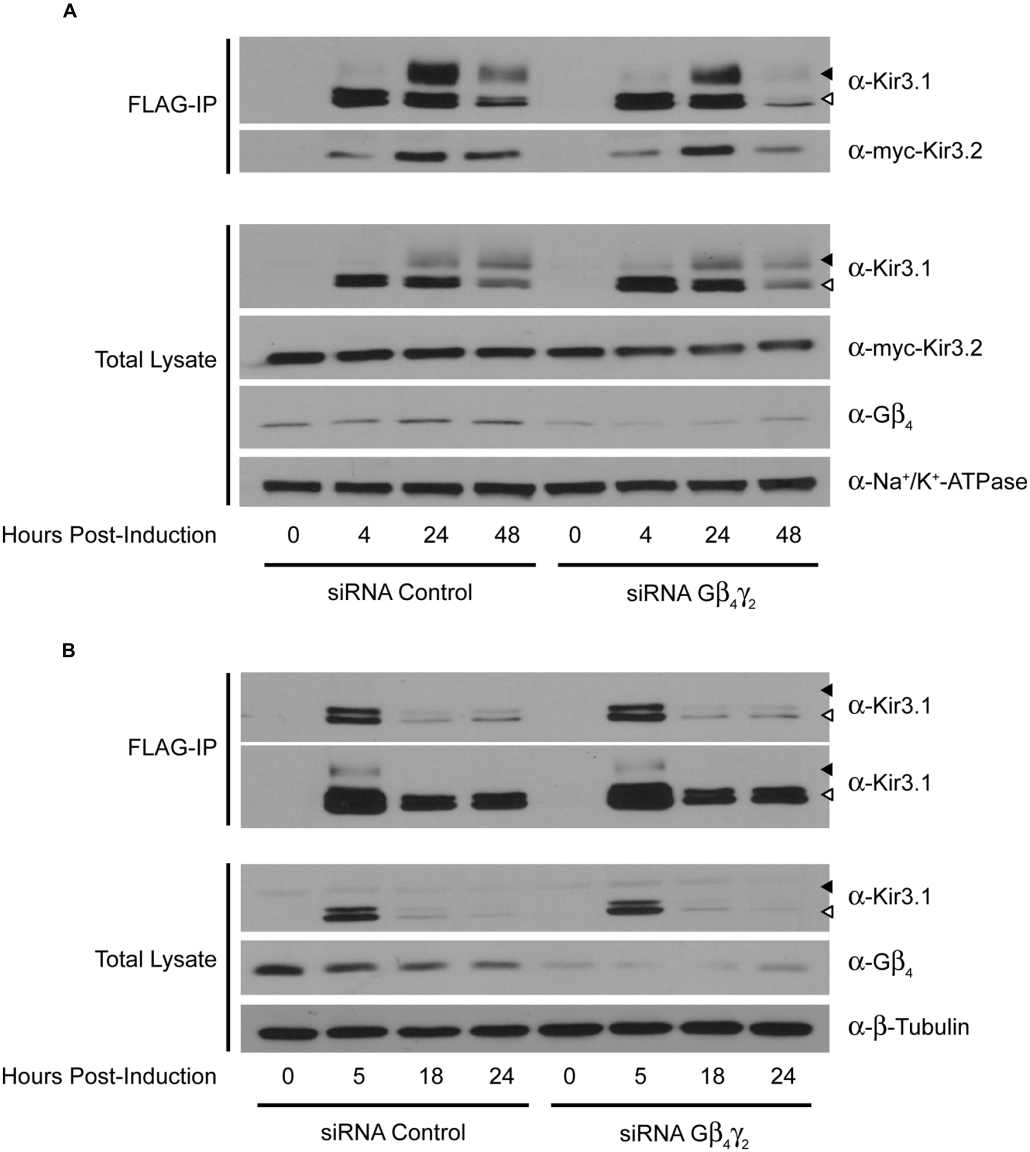
**Double knockdown of Gβ_4_ and Gγ_2_ impairs Kir3.1 stability only in the presence of Kir3.2. (A)** Flp-In Trex FLAG-Kir3.1 cells transfected with myc-Kir3.2 and Gβ_4_/Gγ_2_ or control siRNA were induced to express FLAG-Kir3.1 with 1 μg/mL of tetracycline for 30 min and chased for 0, 4, 24, or 48 h. Cells were lysed and FLAG-Kir3.1 was immunoprecipitated as described above. Upper panel: samples were analyzed by western blot to detect levels of FLAG-Kir3.1 and myc-Kir3.2. Lower panel: total cell lysates showing expression of Kir3.1, Kir3.2, Gβ_4_, and Na^+^/K^+^ ATPase (loading control). **(B)** Experiment was the same as in panel **(A)** except Kir3.2 was not co-expressed and FLAG-Kir3.1 was chased for 0, 5, 18, or 24 h. Two different exposures are shown to highlight effects on both immature (top) and mature (bottom) forms of the channel, respectively. White arrow indicates immature channel proteins while black arrow indicates correctly processed Kir3.1. Results are from a single experiment.

**FIGURE 4:**
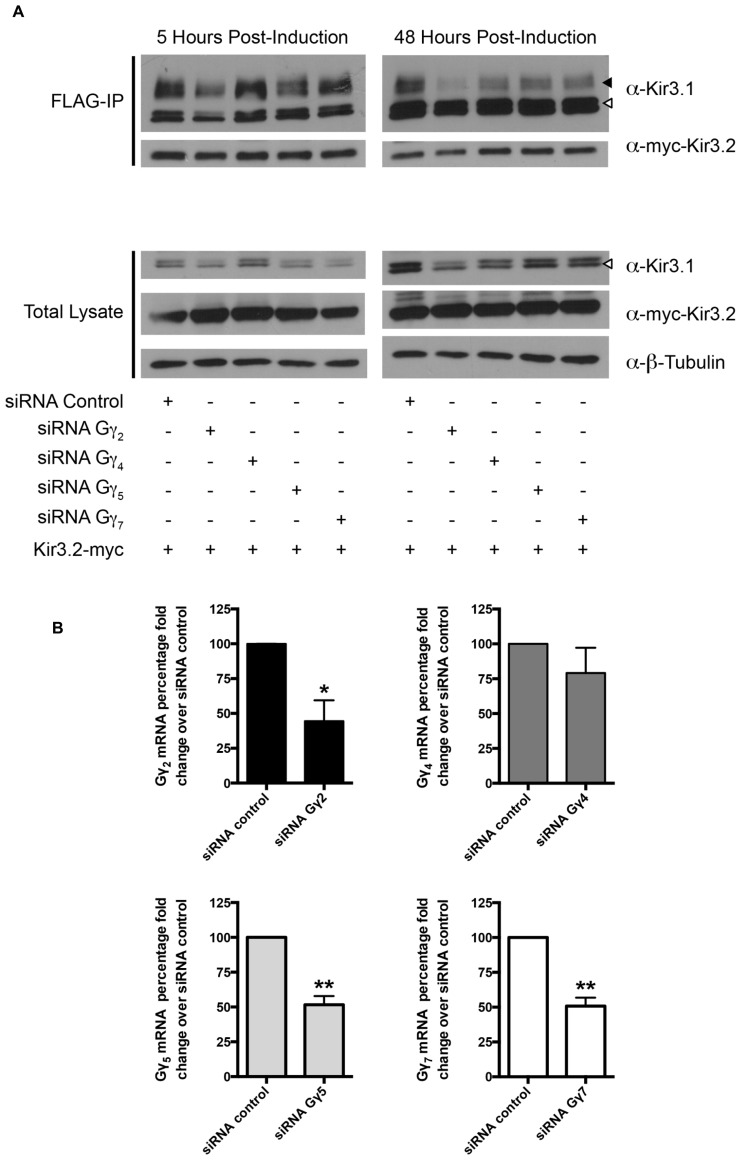
**Knockdown of Gγ isoforms have differential effect on mature Kir3.1 stability. (A)** Flp-In Trex FLAG-Kir3.1 cells were transfected with Gγ2, 4, 5, or 7 targeted siRNA and myc-Kir3.2. The cells were subsequently induced to express FLAG-Kir3.1 with 1 μg/mL of tetracycline for 30 min followed by 5 or 48 h chase times. Cells were lysed and FLAG-Kir3.1 was immunoprecipitated as described above. Upper panel: samples were analyzed by western blot to detect levels of FLAG-Kir3.1 and myc-Kir3.2. Lower panel: total cell lysates showing expression of Kir3.1, Kir3.2, and β-tubulin (as a loading control). Results are shown from a single experiment. White arrow indicates immature channel proteins while black arrow indicates correctly processed Kir3.1. **(B)** Flp-In Trex FLAG-Kir3.1 cells were transfected with 50 nM of specific siRNA targeted against Gγ_2_, Gγ_4_, Gγ_5_, or Gγ_7_. Cells were then lysed in TRI reagent 72 h post-transfection, total RNA was isolated, and the extent of Gγ knockdown was assessed by RT-qPCR using probe specific qPCR assays. Data is depicted as mean ± S.E.M. and is representative of *n* = 3 experiments. Statistical significance was evaluated using an unpaired Student’s *t*-test where * indicates *p* < 0.05 and ** indicates *p* < 0.01.

**FIGURE 5:**
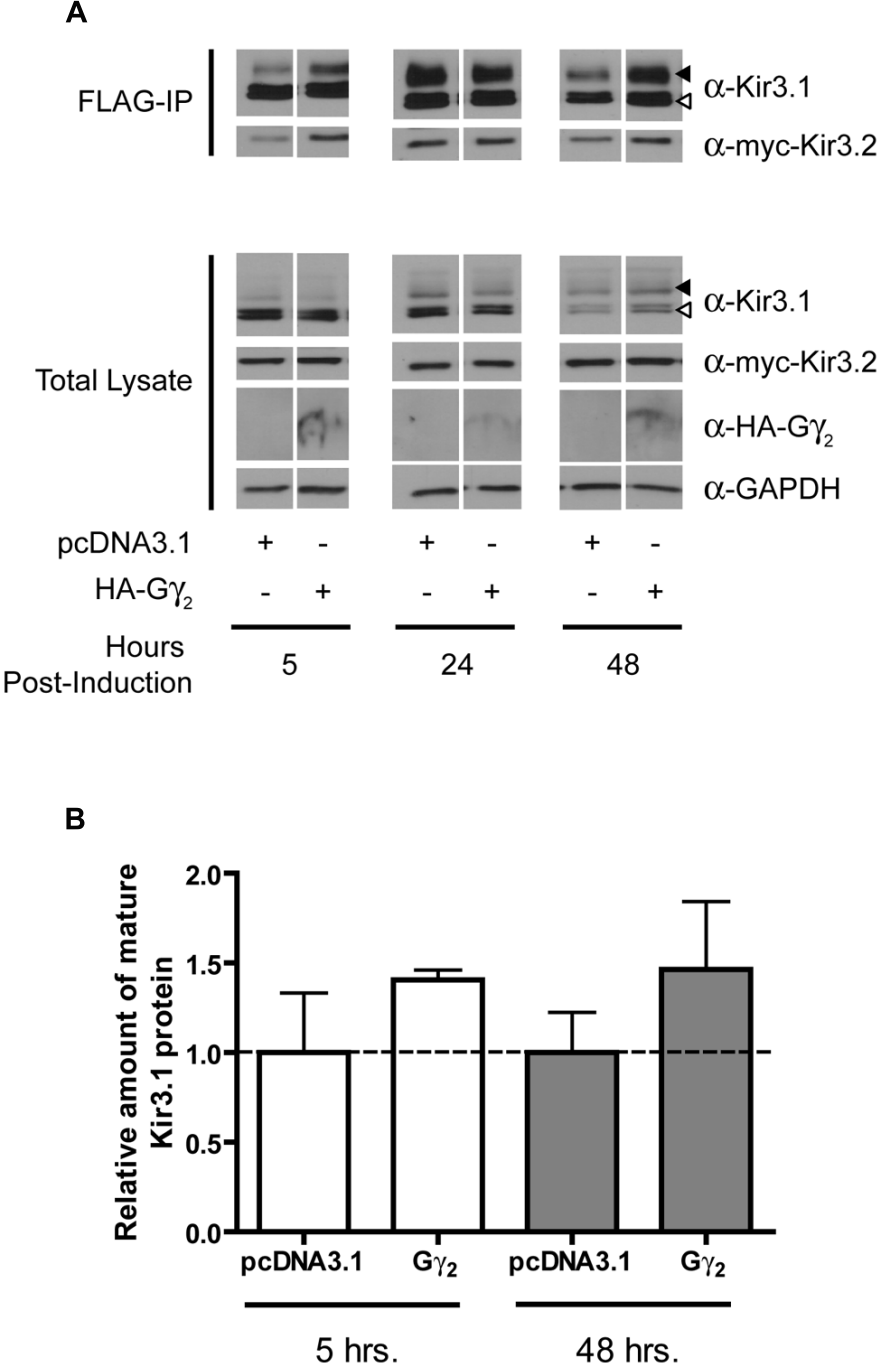
**Overexpression of Gγ2 stabilizes the mature form of Kir3.1. (A)** Flp-In Trex FLAG-Kir3.1 cells were transfected with HA-Gγ_2_ and myc-Kir3.2. The cells were subsequently induced to express FLAG-Kir3.1 with 1 μg/mL of tetracycline for 30 min and chased for 5, 24, or 48 h. Cells were lysed and FLAG-Kir3.1 was immunoprecipitated as described above. Upper panel: samples were analyzed by western blot to detect levels FLAG-Kir3.1 and myc-Kir3.2. Lower panel: total cell lysates showing expression of Kir3.1, Kir3.2, HA-Gγ_2_, and GAPDH (as a loading control). Results are representative of three independent experiments. White arrow indicates immature channel proteins while black arrow indicates correctly processed Kir3.1. **(B)** Densitometric analysis of results obtained above. There is a trend in the effects of Gγ_2_ expression at 48 h, which is reflected in the western blots.

Curiously, we noted that knockdown of Gβ_4_ slightly increased protein expression levels of Gβ_1_ [also observed in a previous study (Krumins and Gilman, 2006)]. The reason for this is unclear at present but we have preliminary data that suggests it may be through transcriptional effects mediated by Gβ_1_ (data not shown). This may also reflect the increased channel maturation in the absence of Gβ_4_, possibly independently of direct competition between the two Gβ subunits. Due to this potential confound of the effect of Gβ_4_ knockdown on Gβ_1_ expression, we next performed an experiment to see if a unique effect of Gβ_4_ could be detected. We knocked down Gβ_4_ in the presence of siRNA against Gγ_2_. In the absence of Gγ_2_, holo-channel stability was also reduced and was not rescued by knockdown of Gβ_4_ (**Figure 3A**). This makes it likely that Gβ_1_ and Gγ_2_ represent the Gβγ pair important for channel maturation. Further, we noted that in the absence of Kir3.2, the stability of the immature forms of Kir3.1 was not affected by knockdown of Gβ_4_γ_2_ (**Figure 3B**). This likely indicates that the functional effect of Gβγ on channel stability requires that Kir3.1 be associated with Kir3.2 and that the “stability clock” modulated by Gβγ begins ticking once the channel is set on a pathway of maturation. Additionally, we tested the effect of knockdown of other Gγ subunits on holo-channel stability. **Figure 3** indicates that knockdown of Gγ_2_ resulted in reduced stability for the Kir3.1/Kir3.2 holo-channel. However, knockdown of Gγ_4_, Gγ_5_, or Gγ_7_ had much more modest effects compared with control siRNA (**Figure 4A**). Knockdown efficiencies of individual Gγ subunits was assessed using RT-qPCR, as endogenous protein expression levels of Gγ isoforms in HEK 293 cells are difficult to visualize using current commercially available anti-Gγ antibodies via western blotting (**Figure 4B**). Changes in expression of the Gγ subunits assessed did not alter expression of Kir3.2 (**Figures 3** and **4**). Finally, overexpression of Gγ_2_ resulted in holo-channels with increased stability (**Figure 5**). Taken together, our results suggest that Gβ_1_γ_2_ serve a unique role in channel trafficking that is distinct from their roles in channel activation, which can possibly be subserved by a number of Gβγ dimers with different isoform specificities.

## DISCUSSION

Kir3 trafficking has been a topic of great interest to understand mechanisms mammalian cells use to regulate their excitability. Though much is known regarding Kir3 trafficking as it progresses along the initial stages of its synthesis and maturation, the role that early interactions with signaling partners such as Gβγ play in channel maturation remain unclear. When expressed alone, recombinant Kir3.1, which contains only one potential site for N-linked glycosylation, migrates as a doublet with a molecular mass of 54 and 56 kDa, with the upper band being the core-glycosylated, immature form of the protein (Krapivinsky et al., 1995a; Kennedy et al., 1999). Upon treatment with either endoglycosidase H, an enzyme that selectively removes N-linked glycosyl moieties from proteins that have not been processed in the Golgi, or endoglycosidase F, an enzyme that non-selectively removes all N-linked sugar residues, the 56 kDa band is virtually abolished, confirming its residence in the ER (Kennedy et al., 1999). Co-expression of Kir3.4 with Kir3.1, results in a fully mature Kir3.1 subunit capable of being properly glycosylated (Kennedy et al., 1999). The role of Kir3.1 glycosylation may be one of quality control, as Kir3.1 subunits containing a mutation preventing glycosylation can still interact with partner subunits and traffic to the plasma membrane yielding typical current amplitudes (Pabon et al., 2000).

Here we show that a number of maturational steps for Kir3.1 are strictly dependent on the presence of Kir3.2. In the absence of Kir3.2, Kir3.1 subunits remain immature and the loss of Gβγ subunits does not affect this outcome. Though most studies have focused on the role of Kir3.4 in the trafficking of Kir3 channels, Kir3.2 subunits have been found to play a similar vital role in forming functional Kir3.1-containing channels at the cell surface. Kir3.2 subunits are able to form both homotetramers as well as heterotetramers with either Kir3.1 or Kir3.3 which can then be targeted to the plasma membrane (Jelacic et al., 2000). Using deletion analysis, it was suggested that an ER export signal found at both the N- and C- terminus of Kir3.2 is vital for the cell surface delivery of Kir3.1/3.2 heterotetramers (Ma et al., 2002). Though no well-defined ER export motif has been characterized for Kir3.3, it has been shown to form tetrameric channels with both Kir3.1 and Kir3.2 subunits (Jelacic et al., 1999). Altogether, Kir3 trafficking from the ER appears to be a highly regulated process, with distinct combinations being available at the plasma membrane or targeted for degradation depending on the needs of the cell. Interestingly, when we inhibit the proteasome with MG-132, Kir3.1 can traffic to the cell surface (P.Z. unpublished observations), demonstrating a likely role for ER-associated degradation (ERAD) in the quality control of unpartnered Kir3.1 subunits.

Much attention has been paid to the specificity of Gβγ dimers in the activation of Kir3 channels. To date, Gβ_5_ stands out as an outlier in the Gβ family in that Gβ_5_-Gγ combinations significantly downregulate channel activation, leading to the suggestion that it acts as a competitive antagonist for Kir3 activation by other Gβγ subunit combinations (Lei et al., 2000, 2003). Interestingly, other Gβγ combinations show similar abilities to activate channels. Some Gβγ selectivity for Kir3.2 has been noted, in that Gβ subtypes 1–3 preferentially interact with this subunit (Robitaille et al., 2009). Most studies have found that any of the four Gβ subunits could be expressed with distantly related Gγ subunits (i.e., Gγ_2_ vs. Gγ_11_) and all combinations could bind and activate Kir3 channels with similar efficacy (Lei et al., 2000).

**FIGURE 6:**
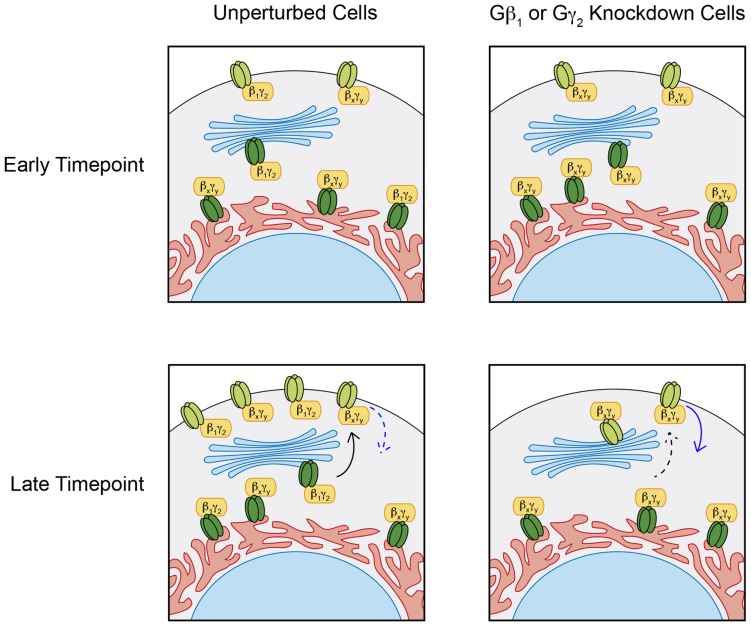
**Distinct Gβγ effects on Kir3 stability.** Under normal conditions, pulse induction of Kir3.1 expression in the presence of Kir3.2 forms heterotetrameric channels of mature Kir3.1 and Kir3.2 (light green), which can traffic to the cell surface and remain expressed at the plasma membrane for up 48 h. siRNA mediated knockdown of Gβ_1_ or Gγ_2_ has negligible effects on immature Kir3.1 channels (dark green) but impairs Kir3.1 maturation and hastens the loss of channel at the cell surface suggesting a stabilizing roles for the Gβ_1_γ_2_ dimer. These effects of Gβ_1_γ_2_ knockdown can be mediated through reduced anterograde trafficking (dashed black arrow) and/or enhanced channel internalization (solid blue arrow). Regardless of the Gβγ pair associated with Kir3 channels in the ER, these are trafficked out to the cell surface. However, those associated with Gβ_1_γ_2_ seem to persist longer than those associated with other Gβγ combinations. This suggests that additional functions exist for some Gβγ combinations, independent of their ability to open the channel in response to GPCR stimulation.

Gβ_1_γ_2_, apart from its role in channel opening shared with other Gβγ dimers, seems to play a unique role in preserving maturing channels from degradation as they transit to the cell surface (summarized in **Figure 6**). Our data indicate that Gβ_1_γ_2_ prolongs the lifetime of the Kir3.1/Kir3.2 heterotetramer, although levels of Kir3.2 are not altered *per se*. This may suggest that Kir3.2 homotetramers might be differentially regulated by other factors although under conditions of bulk overexpression, it is difficult to prove this. Placing the other Kir3 isoforms under the control of inducible promoters using our label-free pulse-chase technique will help resolve these issues. It is clear that the roles Gβγ play in Kir3 activity and regulation are quite complex and likely exceed our current understanding. There are numerous potential Gβγ interacting domains on the tetrameric channel and it is evident that not all of these simply underlie channel activation and gating (i.e., Gβγ-associated channels need not be active until they reach the membrane). Gβγ subunits are involved in channel synthesis, trafficking, plasma membrane stability and degradation (Robitaille et al., 2009; Zylbergold et al., 2010), and it is likely that novel roles beyond mere channel activation will be uncovered in the years to come. Here, we have described some approaches that will allow these issues to be resolved with greater clarity.

## Conflict of Interest Statement

The authors declare that the research was conducted in the absence of any commercial or financial relationships that could be construed as a potential conflict of interest.
